# Simultaneous Detection of Multiple Fish Pathogens Using a Naked-Eye Readable DNA Microarray

**DOI:** 10.3390/s120302710

**Published:** 2012-02-29

**Authors:** Chin-I Chang, Pei-Hsin Hung, Chia-Che Wu, Ta Chih Cheng, Jyh-Ming Tsai, King-Jung Lin, Chung-Yen Lin

**Affiliations:** 1 Aquaculture Division, Fisheries Research Institute, Ministry of Agriculture, 199 Hou-Ih Road, Keelung 20246, Taiwan; E-Mails: ccwu02@mail.tfrin.gov.tw (C.-C.W.); kjlin@mail.tfrin.gov.tw (K.-J.L.); 2 Department of Aquaculture, College of Life Sciences, National Taiwan Ocean University, Pei-Ning Road, Keelung 20224, Taiwan; E-Mail: peihsin.hung@gmail.com; 3 Department of Tropical Agriculture and International Cooperation, National Pingtung University of Science and Technology, 1 Hseuh-Fu Rd., Nei-Pu Hsiang, Pingtung 91201, Taiwan; E-Mail: cheng.tachih@gmail.com; 4 Department of Marine Biotechnology, National Kaohsiung Marine University, 142 Hai-Chuan Road, Kaohsiung 81157, Taiwan; E-Mail: jmtsai@mail.nkmu.edu.tw; 5 Institute of Information Science, Academia Sinica, No. 128 Academia Rd., Sec. 2, Taipei 115, Taiwan; E-Mail: cylin@iis.sinics.edu.tw; 6 Institute of Fisheries Science, College of Life Science, National Taiwan University, No. 1, Roosevelt Rd., Sec 4, Taipei 10617, Taiwan; 7 Division of Biostatistics and Bioinformatics, Institute of Population Health Sciences, National Health Research Institutes, No. 35, Keyan Rd., Zhunan 350, Taiwan

**Keywords:** fish pathogen detection, 16S rDNA, naked-eye reading microarray

## Abstract

We coupled 16S rDNA PCR and DNA hybridization technology to construct a microarray for simultaneous detection and discrimination of eight fish pathogens (*Aeromonas hydrophila*, *Edwardsiella tarda*, *Flavobacterium columnare*, *Lactococcus garvieae*, *Photobacterium damselae*, *Pseudomonas anguilliseptica*, *Streptococcus iniae* and *Vibrio anguillarum*) commonly encountered in aquaculture. The array comprised short oligonucleotide probes (30 mer) complementary to the polymorphic regions of 16S rRNA genes for the target pathogens. Targets annealed to the microarray probes were reacted with streptavidin-conjugated alkaline phosphatase and nitro blue tetrazolium/5-bromo-4-chloro-3′-indolylphosphate, p-toluidine salt (NBT/BCIP), resulting in blue spots that are easily visualized by the naked eye. Testing was performed against a total of 168 bacterial strains, *i.e*., 26 representative collection strains, 81 isolates of target fish pathogens, and 61 ecologically or phylogenetically related strains. The results showed that each probe consistently identified its corresponding target strain with 100% specificity. The detection limit of the microarray was estimated to be in the range of 1 pg for genomic DNA and 10^3^ CFU/mL for pure pathogen cultures. These high specificity and sensitivity results demonstrate the feasibility of using DNA microarrays in the diagnostic detection of fish pathogens.

## 1. Introduction

It may be assumed that fish are continually bathed in an aqueous suspension of microorganisms. Many of the members of the normal microflora of water can be bacterial fish pathogen candidates. *Aeromonas hydrophila*, an etiological agent of fish diseases, is considered as both a primary and secondary pathogen resulting in hemorrhagic septicemia [1]. *Edwardsiella tarda* causes the serious systemic septicemia commonly known as edwardsiellosis, which occurs in cultured Japanese eels [2,3], flounders [4], and tilapias [5]. *Flavobacterium columnare*, which causes gill damage or lesions on the fish body surface has been recognized as a universally occurring pathogen of numerous freshwater fish species, including both coldwater fish [6] and tropical aquarium fish [7]. *Photobacterium damselae* leads to pasteurellosis and infects a wide range of marine species such as white perch [8], yellowtail [9], gilthead seabream [10], sea bass [11], striped jack [12], Japanese flounder [13], and cobia [14]. *Pseudomonas anguilliseptica* causes red spot disease, which is one of the most destructive diseases of pond-cultured eels in Japan [15] and Taiwan [16]. This bacterium has also caused disease outbreaks in European eels in Scotland [17], France [18], and in other farmed fish such as black sea bream [19], salmonid [20], and ayu [21]. Outbreaks of disease caused by *Vibrio anguillarum* represent one of the most commonly occurring examples of vibriosis [22,23]. This pathogen usually produces hemorrhagic septicemia [24], is distributed worldwide, and affects a wide range of fish and shellfish [25–27]. The pathogenic gram-positive cocci *Lactococcus garvieae* and *Streptococcus iniae* usually cause hyperacute and hemorrhagic septicemia in both freshwater and marine aquaculture species such as catfish [28], tilapia [29], trout [30], and yellowtail [31]. These pathogens cause massive mortality and large economic losses in fish farming every year.

The above pathogens that infect cultured species are phylogenetically diverse. Consequently, detection of these pathogens using conventional culture-based microbiological methods is technically demanding and time consuming. The wide diversity of assays combined with frequently fastidious growth conditions make molecular tools such as PCR and DNA microarray better options for detection of fish pathogens. PCR assays have been developed for the rapid detection and identification of microorganisms in clinical samples without the need for further isolation [32,33]. A multiplex PCR (m-PCR) approach that can simultaneously identify several pathogens by the PCR amplicon size using gel electrophoresis has successfully been applied to detect fish and shellfish pathogens [34,35]. However, there are practical limits to PCR assays for detecting multiple pathogens at a time. It is not easy to incorporate more than six primer sets because of the cross-reaction in m-PCR, and the challenges inherent in size discrimination among PCR products by conventional electrophoresis [36]. Subsequent sequencing, which is a relatively costly and laborious process, is often needed to confirm product identity. The new developing method, three oligo (primers + probe) PCR (such as TaqMan® real-time PCR) may overcome the problems. However, it requires more expensive equipment and is suggested to be used in quantitative gene expression and allele discrimination research. Thus, to efficiently screen a complex mixture of sequences from different pathogens, DNA microarray is an excellent candidate.

DNA microarrays are miniaturized microsystems based on the ability of DNA to specifically bind to its complementary sequence in hybridization. Oligonucleotide probes for specific targets are stained at distinct sites on a solid support to which the PCR product is then hybridized and detected [37]. Recent developments in DNA microarray allow parallel hybridizations to occur on the same surface and permit multiple independent detections [38]. In most microarray formats, slides are stained with streptavidin-conjugated fluorophore, and the interaction of the target with specific probes is measured by epifluorescence confocal microscopy using an argon ion laser. On the other hand, precipitation staining methods based on the catalytically induced chromogenic precipitation were applied to the microarray technology. Some commercial products (such as TubeArray™ of Alere Technologies GmbH, Germany and LCD-Array kits of Chipron GmbH, Germany) were developed based on different platforms and chromogenic phosphatase substrates. In this study, the NBT/BCIP (nitro blue tetrazolium/5-bromo-4-chloro-3′-indolylphosphate, p-toluidine salt) microarray system was applied to detection of fish pathogens. In the system, biotin-labelled PCR amplicons are firstly captured on the microarray during hybridization. Then the streptavidin conjugated alkaline phosphatase (Strep-AP) in the staining reagent binds to the biotinylated site. The BCIP in the colorimetric developing reagent reacts to Strep-AP and produces a blue-colored precipitate at the site of enzymatic activity. NBT acts as a co-precipitant agent for the BCIP reaction, forming a dark blue, precisely localized precipitate thus helps to visualize positive spots on the microarray. Here we demonstrate a naked-eye reading microarray system targeting 16S rDNA to identify eight common fish pathogens, obviating the need for expensive fluorescence detection facilities.

## 2. Experimental Section

### 2.1. Bacterial Strains

The strains used in this study are listed in Table 1. These include 26 representative collection strains, 81 isolates of target fish pathogens (belonging to eight species: *A. hydrophila*, *E. tarda*, *F. columnare*, *L. garvieae*, *P. damselae*, *P. anguilliseptica*, *S. iniae* and *V. anguillarum*), and 61 other strains of bacterial species. Strains were grown and maintained following American Type Culture guidelines. In brief, the following organisms were cultured on nutrient agar (incubation temperature/time): *A. hydrophila* (30 °C/24 h), *E. tarda* (37 °C/24 h), *P. aeruginosa* (37 °C/24 h), *P. anguilliseptica* (20 °C/24–36 h), and *Staphylococcus epidermidis* (37 °C/24 h). *A. sobria* (30 °C/24 h) and *A. salmonicida* (26 °C/24–48 h) were cultured on trypticase soy agar. *E. faecalis* (37 °C/24 h), *E. faecium* (37 °C/24 h), *L. garvieae* (30 °C/24–36 h), and *S. iniae* (37 °C/24–48 h) were cultured on brain heart infusion agar. *V. anguillarum* (18 °C/24–48 h) was cultured on enriched nutrient broth. *V. proteolyticus* (26 °C/24–36 h) was cultured on nutrient agar with 3% NaCl. *F. columnare* (20–22 °C/72 h) was cultured on Anacker and Ordal medium. *L. pelagia* (26 °C/24 h), *P. damselae* (26 °C/24–48 h), *V. aestuarianus* (26 °C/24–48 h), *V. alginolyticus* (37 °C/24 h), *V. marinus* (18 °C/36–48 h), *V. salmonicida* (15 °C/48 h), and *V. vulnificus* (30 °C/24 h) were cultured on Marine agar 2216. *V. parahaemolyticus* (25 °C/24 h) was cultured on modified seawater yeast extract agar. *V. harveyi* (26 °C/24 h) was cultured on Photobacterium broth. *Mycobacterium fortuitum* (37 °C/3–5 days) and *M. marinum* (30 °C/5–10 days) were cultured on Middlebrook 7H10 agar with Middlebrook OADC enrichment.

### 2.2. Genomic DNA Preparation

Genomic DNA was extracted from pure cultures using the UltraClean™ Microbial DNA Isolation Kit (MO BIO Laboratories, Inc., Carlsbad, CA, USA), following the manufacturer’s instructions.

### 2.3. Primers and Probes

The specific oligonucleotide probes (Table 2), each consisting of 30 nucleotides, were designed based on the polymorphic regions of 16S rRNA genes of the target pathogens using the unique probe selector program (http://array.iis.sinica.edu.tw/ups/) [39]. The database was made by the retrieved sequences of the 26 reference strains (Table 1) from NCBI GenBank. Alignment of each probe to the 16S rDNA sequences of the species used in this study was then determined using the ClustalW alignment program (DS Gene version 1.5; Accelrys Inc., Tokyo, Japan). Discrimination by certain computer-derived probes was not satisfactory in practice, and therefore, we generated new probes by modifying one or two nucleotides from the original sequences. All oligonucleotides were normalized to a calculated annealing temperature of 65 ± 3 °C and commercially synthesized (Operon Biotechnologies, Inc., USA). Three positive control probes (U735, U1352, and EV71) were used in this study. U735 and U1352 were used to confirm the efficacy of PCR and were designed from the conserved regions of 16S rDNA for eubacteria [40]. EV71 was used to confirm the efficacy of hybridization and was designed from the capsid protein VP1 of the human enterovirus 71 gene (the biotin-labeled EV71 PCR amplicon was incorporated in the hybridization buffer supplied with the kit mentioned below). Thirty poly(A) oligonucleotides were used as the negative control probe. Each probe was chemically synthesized and 5′-amino-modified with the space linker of 15 poly(T) (Operon Biotechnologies, Inc.).

The 16S rDNA universal primers 16S-F and 16S-R were referred to as B27F and U1492R, respectively [41]. The primers were commercially synthesized (Operon Biotechnologies, Inc.) with biotin labeled on the 5′ end to generate biotinylated PCR amplicons that could react with streptavidin-conjugated alkaline phosphatase (Strep-AP) and NBT/BCIP for colormetric signaling on the chips.

### 2.4. Target DNA Amplification

The 16S rDNA of the 168 strains described above was amplified by PCR using universal 16S-F and 16S-R, as described previously [34]. In brief, PCR was performed in a 50 μL reaction mixture containing 0.5 μL of *Taq* DNA polymerase (5 U/μL; Promega), 5 μL of 10× NH_4_ buffer, 2 μL of 10 mM dNTP mix, 10 μL of 10 mM MgCl_2_, 2 μL of 10 μM forward primer 16S-F, 2 μL of 10 μM reverse primer 16S-R, 2 μL of bacterial genomic DNA (100 ng/μL), and 26.5 μL of sterile H_2_O. The cycling protocol was 1 cycle at 94 °C for 5 min, 30 cycles at 94 °C for 2 min, 48 °C for 1.5 min, and 72 °C for 2 min, followed by 1 cycle at 72 °C for 10 min. The resulting approximately 1.5 kbp PCR products were subsequently cloned into the pGEM-T Easy vector (Promega) and sequenced on an ABI 377 automated sequencer (Applied Biosystems, USA) using vector primers. Sequences were compared with GenBank databases using the BLAST program [42]. The sequencing-confirmed 16S rDNA PCR amplicons were then used to determine the positive probes (U735 and U1352) in DNA hybridization using the protocol described below.

### 2.5. Microarray Preparation and Hybridization

**Figure 1.**
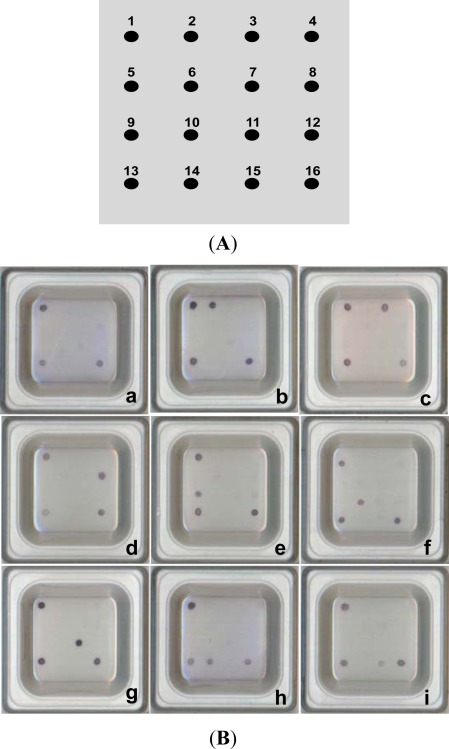
Hybridization and colorization diagram for fish pathogen probes. (**A**) Microarray map. Dots indicate the spotted position of each probe. 1: EV71 (positive control for hybridization); 2: Aehy; 3: Edta; 4: poly(A) (negative control); 5, 6 & 7: blank, with no spotted probes; 8: Flco; 9: Laga; 10: Vian; 11: Phda; 12: blank; 13: U735 (positive control for PCR); 14: Psan; 15: Stin; 16: U1352 (positive control for PCR). (**B**) Detection and typing results on the microarray. a: Positive and negative controls on corners; b: *A. hydrophila*; c: *E. tarda*; d: *F. columnare*; e: *L. garvieae*; f: *V. anguillarum*; g: *P. damselae*; h: *P. anguilliseptica*; i: *S. iniae*.

Spotting 10 μM of each probe to each specific position on the microarray organic polymer substrate (patent no. US-7109024, supplied with the DR. Chip DIY Kit™, DR. Chip Biotechnology, Inc., Miao-Li, Taiwan) was performed using a contact spotting machine (DR. Fast Spot™; DR. Chip Biotechnology, Inc.), and immobilization using a UV crosslinker (Spectroline XLE-1000; Spectronics Corp., New York, USA) with 0.8 J/cm^2^ for 10 min. A schematic diagram of the probe position on the microarray is illustrated in Figure 1(a). Hybridization and colorimetric development were performed using the DR. Chip DIY Kit™ (DR. Chip Biotechnology, Inc.), and all of the reagents including DR. Hyb™ Buffer, Strep-AP, wash buffer, NBT/BCIP and detection buffer were supplied with the Kit. In brief, 15 μL of PCR amplicons were mixed with 200 μL DR. Hyb™ Buffer (DR. Chip Biotechnology, Inc.; 6× SSC, 5× Denhardt’s reagent, 0.5% SDS, 100 μg/mL salmon sperm DNA), denatured in boiling water for 5 min, and immediately chilled on ice for 5 min. The hybridization mixture was transferred to the chip well, incubated at 55 °C with vibration for 60 min, and washed twice with wash buffer (DR. Chip Biotechnology, Inc.; 0.1 M maleic acid, 0.15 M NaCl, pH 7.5). The chip was then added to 0.2 μL Strep-AP (DR. Chip Biotechnology, Inc.; 0.5 μL/mL in blocking buffer) and 200 μL blocking reagent (Roche GmbH, cat. no. 11096176001; 1%). at room temperature (25–35 °C) for 30 min and washed twice again with wash buffer. The colorimetric reaction was implemented by adding 4 μL NBT/BCIP and 196 μL detection buffer (DR. Chip Biotechnology, Inc.; 0.1 M Tris-HCl, 0.1 M NaCl, pH 9.5) to the chip well, developing in the dark at room temperature for 5 min, and washing twice with distilled water. Hybridization results were indicated on the microarray as blue spots that could be read directly by the naked eye.

### 2.6. Specificity of Assay

The specificity of the fish pathogen microarray was evaluated using the genomic DNA extracted from all 168 strains, which was then used as a DNA template for 16S rDNA PCR. Hybridization to the microarray for each PCR amplicon was performed separately using the protocol described above.

### 2.7. Detection Limit of the Microarray

Two approaches for the limit of detection were analyzed. To assess the overall detection limit of the microarray with purified genomic DNA, a serial dilution (100 pg, 10 pg, 1 pg, 100 fg, 10 fg, and 1 fg) of genomic DNA extracted from the eight pathogen collection strains (ATCC7966, ATCC15947, NCIMB2248, MT2055, ATCC19264, ATCC33539, NCIMB2248, and ATCC29178; Table 1) was used as the template for 16S rDNA PCR followed by hybridization to the microarray. To assess the limit of detection of different pathogens in suspension, the eight target pathogens were cultured to the stationary phase in broth medium. Serial dilutions were produced in the range 10^1^–10^9^ CFU/mL in TE-buffer (1 mM Tris-HCl, and 0.5 mM EDTA, pH8). Total DNA of 2 mL of each dilution was extracted using the UltraClean™ Microbial DNA Isolation Kit (MO BIO Laboratories, Inc., Carlsbad, CA, USA), following the manufacturer’s instructions. Two microliters of the extracted DNA was used as a DNA template for PCR and the following microarray assay.

### 2.8. Field Test

#### 2.8.1. Mixed Microbial Cultures and Fish Tissues

To evaluate the availability of the microarray to actual samples, the kidney of tilapia (*Oreochris niloticus × Oreochris aureus*) was used. The kidney samples were examined using the microbiological methods, to confirm that they were germfree. The eight target pathogens were suspended in TE-buffer and adjusted the density to approx. 3 × 10^6^ CFU/mL each. Equal amounts of bacteria were mixed. The kidney tissues were homogenized with the bacterial mixture in a ratio of 1:10 (w/v). Total DNA of 2 mL of the homogenates was extracted using the UltraClean™ Microbial DNA Isolation Kit (MO BIO Laboratories, Inc., Carlsbad, CA, USA), following the manufacturer’s instructions. Two microliters of the extracted DNA was used as a DNA template for PCR and the following microarray assay.

#### 2.8.2. Fishpond Water Samples

The microarray was evaluated for used in surveys of fishpond water sampled from ten local fish farms (seawater, *n* = 5, freshwater, *n* = 5). For microarray assays, total DNA of 2 mL of each water sample was extracted using the UltraClean™ Microbial DNA Isolation Kit (MO BIO Laboratories, Inc., Carlsbad, CA, USA), following the manufacturer’s instructions. Microarray assays were performed separately using the protocol described above with the extracted DNA as a template. In parallel, appropriate dilutions of the water samples were made in sterile 0.85% NaCl solution for bacteriological assays. One hundred microliters of each dilution was plated onto marine agar (BD, USA) for seawater samples or tryptic soy agar (BD, USA) for freshwater samples. The plates were then incubated at 28 °C for 48 h. Ten colonies from the agar plates were randomly picked, purified and identified by the homology of 16S rDNA sequence.

## 3. Results and Discussion

### 3.1. 16S rDNA Amplication and Microarray Hybridization

The 16S rDNA amplicon was obtained by PCR using 16S universal primers 16S-F and 16S-R for each genomic DNA of 168 strains. The resulting approximately 1.5 kbp PCR products were cloned and verified using the sequences retrieved from GenBank databases. For microarray hybridizations, the biotinylated PCR products obtained from pathogen-containing samples were incubated on an organic polymer substrate chip which served the traditional role of dot blot, except that the probe and target positions were reversed. After hybridization and a series of stringency washes, the bound PCR amplicons were reacted with streptavidin-conjugated alkaline phosphatase and NBT/BCIP, resulting in blue spots on the chip. These signals are readily visible to the naked eye, requiring no laser scanning or imaging systems. All PCR amplicons were confirmed by hybridization with either 16S-positive control probes U735 or U1352 on the microarray, which was observed as a blue spot visible to the naked eye (Figure 1).

### 3.2. Probe Specificity

#### 3.2.1. Genomic DNA

A total of 168 strains were used to validate the probe specificity of the microarray. Eight species-specific probes, two PCR-positive control probes, one hybridization-positive control probe, and one negative control probe were finally selected and confirmed for the microarray (Table 2). The identities and gaps for each probe for the 16S rDNA of each representative strain were analyzed using the ClustalW alignment program and are listed in Table 3. In practice tests, only 30 bp DNA probes with fewer than three non-consecutive nucleotide differences and no gap in the 16S rDNA amplicons showed positive results for hybridization and colorization. Good discrimination was evidenced by the fact that all probes consistently distinguished their corresponding target strains with 100% specificity. The hybridization and colorization patterns obtained for the 8 fish pathogens are shown in Figure 1. None of the other 61 strains of bacterial species hybridized to specific probes on the microarray.

#### 3.2.2. Mixed Microbial Cultures and Fish Tissues

**Figure 2.**
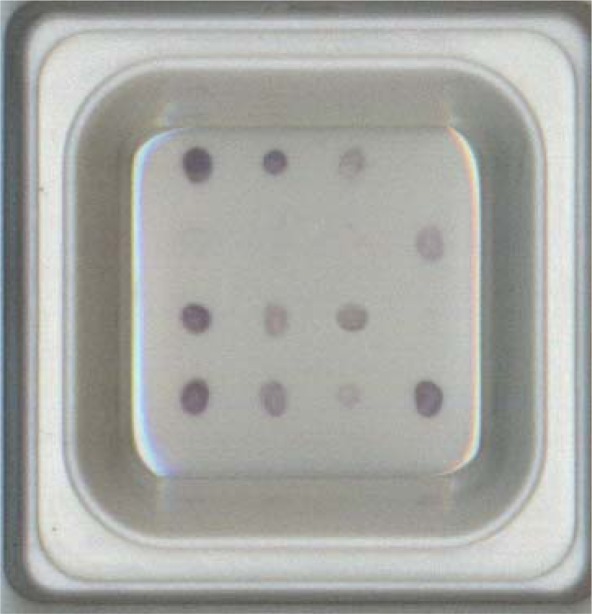
Multiplex hybridization of the 12 probes with the DNA amplicon from the sample with mixtures of fish kidney and the eight target pathogens. The spotted position of each probe was the same with Figure 1.

Results of the preliminary test for the applicability of the microarray is illustrated in Figure 2. The probes for all the 8 pathogens (*A. hydrophila*, *E. tarda*, *F. columnare*, *L. garvieae*, *P. damselae*, *P. anguilliseptica*, *S. iniae* and *V. anguillarum*) gave positive signals. This result demonstrated that the probes designed were specific to their corresponding species.

#### 3.2.3. Fishpond Water Samples

A total of 10 rearing water samples (seawater, *n* = 5, freshwater, *n* = 5) from different local fish farms were analyzed and compared using the microarray and bacteriological methods parallelly. Hybridization results showed that three samples (pond Fw1, Fw2 & Fw4) contained *A. hydrophila*, one sample (pond Fw5) contained *F. columnare*, and one sample (pond Sw2) contained *P. damselae* (Table 4). This finding was consistently confirmed by both 16S rDNA sequencing and bacteriological methods. All samples tested generated positive signals for the control probes U735 and U1352, suggesting that bacteria besides the eight pathogens studied were present. The overall results yielded high accuracy, indicating that this microarray has the ability to detect and discriminate among different pathogens in aquaculture.

The genetic variation in 16S rRNA among species is a subject of debate. Fox *et al*. [43] considered that 16S rRNA sequencing might not be sufficient to guarantee species identity. González *et al*. [44] believed that a high degree of genetic similarity for 16S rRNA genes across species might compromise the specificity of PCR detection. It will detect different species by using this 16S rRNA, indicating more common instead of more specificity. The strategies of probe design and microarray technology we used in this study can overcome the obstacles mentioned above. Firstly, with assistance from the Unique Probe Selector program [39], we deliberately designed specific probes based on polymorphic regions of the 16S rRNA gene with as high a degree of variation as possible. Secondly, the length of each probe was designed to be as short as 30 nucleotides. According to a hybridization rule-of-thumb of 10–15% [38,45,46], a DNA duplex will form between targets and their complementary probes if genetic dissimilarity is <10–15%, *i.e*., three nucleotides of the 30-mer probe in our case. A duplex between targets (16S PCR amplicons, ∼1,500 bp) and probes (30 bp each) is less likely to form when dissimilarity exceeds three bases, particularly when base mismatches are distributed systematically [47]. Furthermore, hybridization stringency is positional- and context-sensitive. If mismatches occur at the terminal ends of the probe, the effects will be less compared with when they occur throughout the probe sequence [48]. These three general rules for DNA duplex formation were applied appropriately to probe design in this study. For example, the original sequence of the probe Phda (Table 2), as calculated from the Unique Probe Selector program, was 5′-cgggcctctcgcgtcaggattagcccaggt-3′, which is 100% identical to that of *P. damselae*. However, there were only two mismatches between the original Phda probe and the 16S PCR amplicons from the phylogenetically related *Vibrio* spp., e.g., *V. marinus*, *V. proteolyticus*, *V. salmonicida*, and *V. vulnificus*, which led to false-positive hybridization results. Therefore we revised two nucleotides from the probe Phda (G changed to T, T changed to A at positions 23 and 30, respectively, Table 2). The revised Phda (5′-cgggcctctcgcgtcaggattaTcccaggA-3′) showed two nucleotide mismatches to the 16S rDNA of *P. damselae*, but one mismatch occurred at the 3′ end of the probe with less effect on hybridization. In comparison with the phylogenetically related *Vibrio* spp., four nucleotides differed from their 16S rRNA genes (Table 3) and at least three mismatches were distributed throughout the probe sequences to interrupt duplex formation. Using a similar strategy, the microarray we constructed was demonstrated as discriminating the eight target pathogens from 26 ecologically and/or phylogenetically related bacteria (Table 1), some of which were not distinguishable by 16S PCR and electrophoresis.

### 3.3. Detection Limit of the Microarray

#### 3.3.1. Genomic DNA

Under ideal conditions, genomic DNA was extracted from the eight purified pathogenic collection strains and serially diluted (100 pg, 10 pg, 1 pg, 100 fg, 10 fg, and 1 fg) as the template for 16S PCR to test the detection limit of the microarray. Positive signals were generated from DNA dilutions 1 pg (Table 5). The lowest detected concentration of genomic DNA in the microarray was 0.1 pg for *A. hydrophila*. Visible bands of 16S PCR amplicons were not observed at this concentration by standard gel electrophoresis. However, for other strains, DNA <1 pg generated either ambiguous or no signals. Therefore, we decided to use 1 pg as the DNA detection limit for this microarray. All 81 strains of the 8 target species were tested and were shown to have been detected successfully at this concentration.

#### 3.3.2. Bacteria from Pure Culture

**Figure 3.**
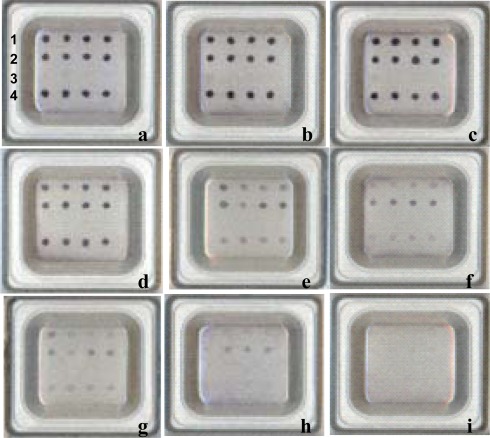
Detection limit assay on the microarray with serially diluted cultures of *Edwardsiella tarda.* Row 1: dotting with the probe U735. Row 2: dotting with the probe Edta. Row 3: dotting with the probe poly(A). Row 4: dotting with the probe U1352. *E. tarda* suspensions in serial dilutions were used as samples for microarray detection. Bacterial concentrations (CFU/mL): (**a**) 4 × 10^9^; (**b**) 4 × 10^8^; (**c**) 4 × 10^7^; (**d**) 4 × 10^6^; (**e**) 4 × 10^5^; (**f**) 4 × 10^4^; (**g**) 4 × 10^3^; (**h**) 4 × 10^2^; (**i**) 4 × 10^1^. Positive signals resulted from four independent PCR amplicons.

For the purpose of directly detecting pathogens in suspension samples, serially diluted cultures of the eight target strains were tested using the microarray. All target pathogens were detectable at concentrations in the range 10^3^–10^4^ CFU/mL (Table 6). For example, *E. tarda* was selected as representative and serial dilutions prepared in the range 4 × 10^1^–4 × 10^9^ CFU/mL. The detection limit was as low as 4 × 10^3^ CFU/mL (Figure 3).

Several factors could result in the detection of false-negatives on microarray. Direct capturing of 16S rRNA with surface-immobilized oligonucleotides is strongly influenced by inaccessible secondary structures, which may encompass some of the most variable binding sites of target molecules [49]. The addition of “helper” oligonucleotides permitted greater accessibility of the corresponding target region for probe hybridization and had a clear impact on the signal intensity of particular probes [50]. However, the application of helper oligonucleotides resulted in a dramatic increase in overall signal intensity, including the mismatch controls [51]. Another parameter that has to be considered in the context of signal limitation is steric hindrance. When hybridizing on a solid support, the binding efficiency of target molecules may be reduced by unfavourable steric interactions mediated by the solid matrix [52]. Peplies *et al.* [51] indicated that the addition of 12-mer and 18-mer poly(A) spacers to the probe sequence can mitigate structural inhibition. These authors found a linear correlation between spacer length and measured signal intensity even without the addition of helper oligonucleotides. Since the molar concentration of PCR product is very high, the concentration of target molecules using our detection protocol was much greater than that by direct detection of 16S rRNA on microarray [50], we decided not to take any risk by increasing the non-specific signal. Therefore, we added 15-mer poly(T) spacer to the 5′-end of each probe but added no helper oligonucleotides to the hybridization buffer. With hybridization conditions being considered adequate in our study, false-negative signals in the testing of 81 strains of target species did not occur.

The microarray system described herein could detect as little as 1 pg of purified genomic DNA, which is equivalent to 200–250 cells. This is not as sensitive as that previously reported in the literature, where lower detection limits were observed, e.g., 675 fg of *Yersinia ruckeri* DNA that was amplified with 16S universal primers [36] and 10 fg of *Bacillus anthracis* DNA that was amplified with species-specific primers [53]. Not surprisingly, regular microarray systems detect fluorescent signals of hybridization by laser reader, which is 10- to100-fold more sensitive than our naked-eye reading system, especially for small amounts of target molecules in samples. However, with our system we demonstrated the limit of detection with 10^3^–10^4^ CFU/mL of target pathogen in serially diluted suspensions (Figure 3). These results are comparable to the detection limit of the fluorescent-labeled microarray system. Zhou *et al*. [54] reported that in 10^2^–10^5^ CFU/mL serial dilutions of *S. aureus*, the optimal positive signal was obtained with 10^4^ CFU/mL. In studies by Maynard *et al.* [55], using a combination of PCR followed by microarray hybridization, the detection limit for *Salmonella enterica* was estimated to be on the order of 10^4^ CFU/mL. Agreement between these data indicates that when the amount of nucleic acid is not limiting, the economic naked-eye reading microarray system may prove very valuable as a tool for discriminating multiple pathogens in aquaculture.

## 4. Conclusions

The need to instantaneously monitor pathogen threats in aquaculture has led to the development of simultaneous detection systems. Oligonucleotide microarray, combining PCR technology with hybridization of the resulting amplification products, and post hybridization image processing have produced extremely powerful tools for pathogen detection, differentiation, and identification. In this report, we used this technology to design a DNA microarray containing specific oligonucleotide probes for the 16S rDNA polymorphic regions of eight aquacultural candidate pathogens. It was demonstrated to discriminate the 8 target pathogens from 26 ecologically or phylogenetically related bacteria, some of them were not distinguishable by 16S PCR and electrophoresis. Furthermore, we chose a naked-eye reading microarray system. The resulting signals are readily visible to the naked eye, requiring no laser scanning or imaging systems. The entire microarray manipulation time was less than 2 h, equivalent to the time needed for gel electrophoresis. This DNA microarray is well suited for detection of multiple fish pathogens in aquaculture.

## Figures and Tables

**Table 1.** Bacterial strains used in this study.

**Species**	**Number of strains from different sources**	**Collection strain ^a^**	**Total number**
*Aeromonas hydrophila*	1 ^a^, 18 ^b^	ATCC7966	19
*A. sobria*	1 ^a^	ATCC43979	1
*A. salmonicida*	1 ^c^	MT423	1
*Edwardsiella tarda*	1 ^a^, 30 ^b^	ATCC15947	31
*Enterococcus faecalis*	1 ^a^	ATCC19433	1
*E. faecium*	1 ^a^, 1 ^b^	ATCC19434	2
*Flavobacterium columnare*	1 ^d^	NCIMB2248	1
*Lactococcus garvieae*	1 ^c^, 14 ^b^	MT2055	2
*L. pelagia*	1 ^a^	ATCC25916	1
*Mycobacterium fortuitum*	1 ^a^	ATCC19709	1
*M. marinum*	1 ^a^	ATCC927	1
*Photobacterium damselae* subsp. *damselae*	1 ^a^, 3 ^b^	ATCC33539	4
*P. damselae* subsp. *piscicida*	1 ^a^, 13 ^b^	ATCC51736	14
*Pseudomonas aeruginosa*	1 ^a^, 1 ^b^	ATCC10145	2
*P. anguilliseptica*	1 ^d^	NCIMB2248	1
*Staphylococcus epidermidis*	1 ^a^, 1 ^e^	ATCC12228	2
*Streptococcus iniae*	1 ^a^, 1 ^b^	ATCC29178	2
*Vibrio aestuarianus*	1 ^a^	ATCC35048	1
*V. alginolyticus*	1 ^a^, 26 ^b^	ATCC17749	27
*V. anguillarum*	1 ^a^, 4 ^b^	ATCC19264	5
*V. harveyi*	1 ^a^, 22 ^b^	ATCC14126	23
*V. marinus*	1 ^a^	ATCC15382	1
*V. parahaemolyticus*	1 ^a^, 4 ^b^	ATCC27969	5
*V. proteolyticus*	1 ^a^	ATCC15338	1
*V. salmonicida*	1 ^a^	ATCC43839	1
*V. vulnificus*	1 ^a^, 4 ^b^	ATCC27562	5

a American Type Culture Collection (ATCC);

b Fisheries Research Institute, Taiwan (FRI),

c FRS Marine Laboratory, UK (MT);

d NCIMB, National Collection of Industrial, Marine and Food Bacteria, UK (NCIMB);

e Bioresource Collection and Research Center, Taiwan (BCRC).

**Table 2.** Oligonucleotides used in this study.

**Oligo nucleotide**	Sequence *	**Tm (°C)**	**Target organism**	**Accession no.**
**Probe**				

Aehy	ggttAatgcctaatacgtatcaactgtgac	62.21	*A. hydrophila*	DQ207728
Edta	ctcatgccatcaTatgaacccagatgggat	62.62	*E. tarda*	DQ233654
Flco	ccctgttgctagttgccagcgagtcatgtc	65.01	*F. columnare*	AY095342
Laga	tcgccaacccgcgagggtgcgctaatctct	67.68	*L. garvieae*	AY699289
Phda	cgggcctctcgcgtcaggattaTcccaggA	65.50	*P. damselae*	AY147861
Psan	ccgttggaatccttgagattttagtggcgc	66.29	*P. anguilliseptica*	HM103328
Stin	ggtgttaggccctttccggggcttagtgcc	66.99	*S. iniae*	AF335572
Vian	tgacatctacagaatcctgcggagacgcgg	68.45	*V. anguillarum*	X16895
U735	actgaggtgcgaaagcgtggggagcaaaca	65.28	Eubacteria	AF233451
U1352	tgaatacgttcccgggccttgtacacaccg	65.83	Eubacteria	AF233451
EV71	atgaagcatgtcagggcttggatacctcg	63.17	Human enterovirus 71	HQ283840
poly(A)	aaaaaaaaaaaaaaaaaaaaaaaaaaaaaa			

**Primer**				

16S-F	agagtttgatcatggctcag	49.73	Eubacteria	AF233451
16S-R	ggttaccttgttacgactt	46.77	Eubacteria	AF233451

* Nucleotides designed differently from the original sequences are shown in uppercase.

**Table 3.** Identities and gaps derived from ClustalW for each probe to the 16S rDNA of each representative strain used in this study.

**Probe**	**Identity (upper row)/gap (lower row)**							
	**Aehy**	**Edta**	**Flco**	**Laga**	**Phda**	**Psan**	**Stin**	**Vian**
**Organism**								

*A. hydrophila*	29/30 *	19/30	21/30	18/30	21/30	20/30	21/30	24/30
	**0/30**	0/30	0/30	0/30	0/30	2/30	9/30	0/30
*A. sobria*	21/30	22/30	21/30	18/30	23/30	20/30	19/30	23/30
	0/30	0/30	0/30	0/30	0/30	2/30	1/30	0/30
*A. salmonicida*	26/30	22/30	21/30	18/30	22/30	20/30	21/30	23/30
	0/30	0/30	0/30	0/30	0/30	2/30	09/30	0/30
*E. tarda*	19/30	**29/30**	22/30	18/30	23/30	23/30	19/30	23/30
	1/30	**0/30**	2/30	0/30	2/30	11/30	3/30	0/30
*E. faecalis*	22/30	18/30	21/30	22/30	16/30	19/30	18/30	21/30
	7/30	0/30	1/30	0/30	0/30	0/30	0/30	2/30
*E. faecium*	22/30	18/30	22/30	23/30	16/30	19/30	21/30	20/30
	7/30	0/30	1/30	0/30	0/30	0/30	3/30	4/30
*F. columnare*	22/30	23/30	**30/30**	19/30	23/30	20/30	22/30	22/30
	3/30	5/30	**0/30**	1/30	5/30	4/30	7/30	2/30
*L. garvieae*	20/30	18/30	21/30	**30/30**	20/30	19/30	19/30	21/30
	10/30	1/30	1/30	**0/30**	6/30	0/30	1/30	2/30
*L. pelagia*	18/30	23/30	25/30	20/30	25/30	22/30	19/30	21/30
	1/30	2/30	1/30	0/30	0/30	11/30	3/30	3/30
*M. fortuitum*	20/30	18/30	20/30	20/30	19/30	20/30	20/30	22/30
	2/30	2/30	0/30	3/30	1/30	2/30	1/30	1/30
*M. marinum*	20/30	19/30	20/30	21/30	20/30	23/30	20/30	18/30
	4/30	2/30	0/30	3/30	1/30	8/30	1/30	0/30
*P. damselae*	20/30	23/30	20/30	19/30	**28/30**	22/30	21/30	21/30
	1/30	2/30	1/30	0/30	**0/30**	11/30	7/30	0/30
*P. aeruginosa*	22/30	22/30	20/30	23/30	20/30	24/30	22/30	22/30
	1/30	0/30	0/30	0/30	0/30	0/30	3/30	2/30
*P. anguilliseptica*	23/30	22/30	20/30	23/30	19/30	**30/30**	20/30	22/30
	1/30	0/30	0/30	0/30	0/30	**0/30**	4/30	2/30
*S. epidermidis*	20/30	19/30	21/30	19/30	22/30	17/30	22/30	22/30
	2/30	0/30	2/30	0/30	6/30	0/30	0/30	2/30
*S. iniae*	22/30	23/30	22/30	22/30	21/30	17/30	**30/30**	21/30
	10/30	6/30	1/30	0/30	4/30	0/30	**0/30**	2/30
*V. aestuarianus*	18/30	23/30	25/30	20/30	25/30	23/30	19/30	26/30
	1/30	2/30	1/30	0/30	0/30	1/30	3/30	0/30
*V. alginolyticus*	22/30	23/30	25/30	21/30	25/30	22/30	19/30	21/30
	5/30	2/30	1/30	0/30	0/30	11/30	3/30	0/30
*V. anguillarum*	18/30	23/30	25/30	18/30	25/30	22/30	20/30	**30/30**
	1/30	2/30	1/30	0/30	0/30	11/30	4/30	**0/30**
*V. harveyi*	24/30	23/30	20/30	21/30	25/30	21/30	19/30	21/30
	10/30	2/30	1/30	0/30	0/30	10/30	3/30	3/30
*V. marinus*	20/30	19/30	21/30	18/30	26/30	23/30	21/30	22/30
	6/30	0/30	1/30	0/30	0/30	1/30	4/30	1/30
*V. parahaemolyticus*	18/30	23/30	25/30	20/30	25/30	22/30	19/30	21/30
	1/30	2/30	1/30	0/30	0/30	11/30	3/30	0/30
*V. proteolyticus*	23/30	24/30	25/30	20/30	26/30	22/30	20/30	22/30
	10/30	2/30	1/30	0/30	0/30	11/30	4/30	0/30
*V. salmonicida*	23/30	19/30	21/30	18/30	26/30	22/30	19/30	22/30
	7/30	0/30	1/30	0/30	0/30	11/30	2/30	1/30
*V. vulnificus*	22/30	24/30	25/30	20/30	26/30	22/30	19/30	25/30
	7/30	2/30	1/30	0/30	0/30	11/30	3/30	0/30

* Features in boldface represent the positive results for hybridization and color development.

**Table 4.** Hybridization results of fishpond water samples assayed by the microarrays.

**Probe Sample**	**Hybridization signal**											
	**Aehy**	**Edta**	**Flco**	**Laga**	**Phda**	**Psan**	**Stin**	**Vian**	**U735**	**U1352**	**EV71**	**poly(A)**
Seawater pond												
Sw1	−	−	−	−	−	−	−	−	+	+	+	−
Sw2	−	−	−	−	+	−	−	−	+	+	+	−
Sw3	−	−	−	−	−	−	−	−	+	+	+	−
Sw4	−	−	−	−	−	−	−	−	+	+	+	−
Sw5	−	−	−	−	−	−	−	−	+	+	+	−

Freshwater pond												

Fw1	+	−	−	−	−	−	−	−	+	+	+	−
Fw2	+	−	−	−	−	−	−	−	+	+	+	−
Fw3	−	−	−	−	−	−	−	−	+	+	+	−
Fw4	+	−	−	−	−	−	−	−	+	+	+	−
Fw5	−	−	+	−	−	−	−	−	+	+	+	−

	**Bacterial composition** (number of species identified by 16S rDNA homology)											

Sw1	*Alteromonas* sp. (2), *Pseudoalteromonas* sp. (3), *Rhodobacteraceae bacterium* (3), *Vibrio alginolyticus* (2)											

Sw2	*Photobacterium damselae* (2), *Pseudoalteromonas* sp. (2), *Vibrio fortis* (1), *Vibrio harveyi* (1), *Vibrio* sp. (4)											

Sw3	*Gamma proteobacterium* (3), *Maribacter dokdonensis* (1), *Pseudoalteromonas* sp. (2), *Pseudomonas* sp. (1), *Roseobacter gallaeciensis* (1), *Tenacibaculum* sp. (2)											

Sw4	*Vibrio alginolyticus* (1), *Vibrio harveyi* (6), *Vibrio* sp. (3)											

Sw5	*Alteromonas* sp. (2), *Gamma proteobacterium* (3), *Pseudoalteromonas* sp. (2), *Rhodobacteraceae bacterium* (1), *Sulfitobacter* sp. (2)											

Fw1	*Aeromonas hydrophila* (3), *Aeromonas* sp. (3), *Citrobacter freundii* (2), *Plesiomonas shigelloides* (1), *Pseudomonas* sp. (1)											

Fw2	*Aeromonas hydrophila* (3), *Aeromonas sobria* (1), *Bacillus* sp. (3), *Citrobacter freundii* (1), *Citrobacter* sp. (2)											

Fw3	*Aeromonas sobria* (2), *Aeromonas* sp. (1), *Citrobacter* sp. (3), *Plesiomonas shigelloides* (3), *Pseudomonas* sp. (1)											

Fw4	*Aeromonas hydrophila* (1), *Bacillus cereus* (3), *Bacillus subtilis* (3), *Plesiomonas shigelloides* (3)											

Fw5	*Aeromonas sobria* (1), *Bacillus subtilis* (1), *Citrobacter freundii* (1), *Citrobacter* sp. (2), *Flavobacterium columnare* (1), *Plesiomonas shigelloides* (2), *Pseudomonas* sp. (2)											

**Table 5.** Detection limit of the microarray with serially diluted genomic DNA.

**Species**	**Genomic DNA (pg)**					
	**100**	**10**	**1**	**0.1**	**0.01**	**0.001**
*Aeromonas hydrophila*	+	+	+	+	−	−
*Edwardsiella tarda*	+	+	+	±	−	−
*Flavobacterium columnare*	+	+	±	−	−	−
*Lactococcus garvieae*	+	+	±	−	−	−
*Photobacterium damselae*	+	+	+	−	−	−
*Pseudomonas anguilliseptica*	+	+	+	±	−	−
*Streptococcus iniae*	+	+	±	−	−	−
*Vibrio anguillarum*	+	+	+	±	−	−

+ positive signal; − negative signal; ± weak or ambiguous signal.

**Table 6.** Detection limit of the microarray with serially diluted bacterial cultures.

**Species**	**Bacterial counts (CFU/mL)**					
	**>10^6^**	**10^5^**	**10^4^**	**10^3^**	**10^2^**	**10^1^**
*Aeromonas hydrophila*	+	+	+	+	±	−
*Edwardsiella tarda*	+	+	+	+	±	−
*Flavobacterium columnare*	+	+	+	±	−	−
*Lactococcus garvieae*	+	+	+	+	±	−
*Photobacterium damselae*	+	+	+	±	−	−
*Pseudomonas anguilliseptica*	+	+	+	+	±	−
*Streptococcus iniae*	+	+	+	±	−	−
*Vibrio anguillarum*	+	+	+	+	−	−

+ positive signal; − negative signal; ± weak or ambiguous signal.
